# Association of daily coffee and tea consumption and metabolic syndrome: results from the Polish arm of the HAPIEE study

**DOI:** 10.1007/s00394-014-0789-6

**Published:** 2014-11-04

**Authors:** Giuseppe Grosso, Urszula Stepaniak, Agnieszka Micek, Roman Topor-Mądry, Hynek Pikhart, Krystyna Szafraniec, Andrzej Pająk

**Affiliations:** 1 Department of Clinical and Molecular Biomedicine, Section of Pharmacology and Biochemistry, University of Catania, V.le A. Doria 6, 95125 Catania, Italy; 2 Department of Epidemiology and Population Studies, Jagiellonian University Medical College, Kraków, Poland; 3 Department of Epidemiology and Public Health, University College London, London, UK

**Keywords:** Coffee, Tea, Metabolic syndrome, Blood pressure, Waist circumference, Dyslipidemia, Hyperglycemia

## Abstract

**Purpose:**

The aim of this study was to evaluate whether daily consumption of coffee and tea was associated with components and prevalence of metabolic syndrome (MetS) in the Polish arm of the Health, Alcohol and Psychosocial factors In Eastern Europe cohort study.

**Methods:**

A cross-sectional population-based survey including 8,821 adults (51.4 % female) was conducted in Krakow, Poland. Coffee and tea consumption was evaluated using food frequency questionnaires. MetS was defined according to the International Diabetes Federation definition. Linear and logistic regression models were performed to estimate odds ratios and confidence intervals.

**Results:**

Among high coffee and tea consumers (3 or more cups/day), high prevalence of female gender, young age, medium–high educational and occupational level, high total energy intake, and smoking habit were found. High coffee drinkers had lower BMI, waist circumference, systolic and diastolic blood pressure, triglycerides, and higher HDL cholesterol than those drinking less than 1 cup/day. In contrast, high tea consumers had lower BMI, waist circumference, but not diastolic blood pressure, which was higher than low drinkers. After adjusting for potential confounding factors, both higher coffee and tea consumption were negatively associated with MetS (OR 0.75, 95 % CI 0.66, 0.86 and OR 0.79, 95 % CI 0.67, 0.92, respectively). Among specific components of MetS, high coffee consumption was negatively associated with waist circumference, hypertension, and triglycerides, whereas tea consumption with central obesity and fasting plasma glucose in women, but not in men.

**Conclusions:**

Coffee and tea consumption was negatively associated with MetS and some of its components.

## Introduction

Metabolic syndrome (MetS) is defined by the presence of a group of cardiovascular risk factors, such as hyperglycemia, dyslipidemia, hypertension, and abdominal obesity, which clustered together are associated with greater cardiovascular disease risk [1, 2]. The prevalence of MetS has increased over recent decades, reaching alarming rates worldwide [3, 4]. However, there are important differences between regions, which could be attributed to diet and lifestyle that specifically differ by country [5, 6]. A protective effect is attributable, at least in part, to the contents of plant-derived foods and bioactive phytochemicals in the diet. Indeed, the components of MetS have been inversely associated with dietary pattern including polyphenol-rich foods, such as fruit and vegetables as well as olive oil and red wine [7].

Recently, increasing experimental and epidemiological studies pointed out the possible beneficial effects of coffee and tea on cardiovascular disease risk [8]. These two beverages are among the most consumed worldwide, with higher amount, especially in non-Mediterranean countries [9, 10]. Epidemiological studies showed that regular coffee intake improved some components of the MetS, including hypertension and diabetes mellitus [11, 12]. Tea consumption has been reported to protect against cardiovascular disease (CVD) by reducing blood pressure, blood glucose levels, and body weight [13]. The beneficial effects of tea and coffee consumption could be explained by their high content of vitamins and polyphenols, which are suggested to be negatively associated with chronic diseases [14, 15]. The potential health benefits depend on their antioxidant and antiinflammatory bioactivity, which may contribute to their protective role against CVD [16]. A growing number of studies have suggested that coffee and tea polyphenols may be useful for the prevention of obesity and MetS [17, 18]. Although results of experimental studies are not conclusive, the effects of tea and coffee consumption should be evaluated in the observational studies, since acute administration of caffeinated beverages in clinical trials leads to inconsistent or detrimental effects on health [19].

Studies regarding the association of coffee and tea consumption with MetS are scarce. Some studies conducted in Japan demonstrated a significant inverse correlation between coffee intake and MetS [20–22]. Other studies conducted in the European setting failed to demonstrate any relation [23–25], whereas another study conducted in a Mediterranean area reported a favorable effect [26]. However, investigations conducted on large and well-established nutritional cohorts are still lacking.

The aim of this study was to evaluate whether daily consumption of coffee and tea was independently associated with components and prevalence of MetS in a large cohort from the Krakow town in Poland. The association of coffee and tea consumption with multiple markers for MetS, including body mass index (BMI), waist circumference (WC), fasting plasma glucose (FPG), total cholesterol, HDL-cholesterol (HDL-c), LDL-cholesterol (LDL-c), serum triglycerides, and systolic and diastolic blood pressure (SBP and DBP, respectively), was explored.

## Subjects and methods

### Study population

Subjects were participants of the Polish arm of the Health, Alcohol and Psychosocial factors In Eastern Europe (HAPIEE) study, which was a prospective cohort study aimed to investigate the determinants of CVD and other chronic conditions in Central and Eastern Europe. The study protocol with the rationale, design, and methods has been described in detail elsewhere [27]. Briefly, a random sample of 10,728 subjects (aged 45–69 year) was recruited at the baseline survey conducted in 2002–2005 (response ratio of 59 %) in the urban area of Krakow, Poland. The survey involved completion of structured questionnaires and an examination in clinic. The questionnaires covered health, medical history, health behavior, socioeconomic circumstances, psycho-social factors, and diet. The participants provided written informed consent, and the study protocol was approved by the ethics committee at University College London, UK and by the bioethics committee of the Jagiellonian University (no. KE/99/03/B/284 2).

Among participants who attended the clinical visit (*n* = 9,050), those with missing outcome measures, with incomplete (more than 50 % of answers missing) or incongruent (energy intake <500/>4,000 kcal/day for females and <800/>5,000 kcal/day for males) data regarding dietary information were excluded, resulting in a final sample of 8,821 adults (51.4 % female).

### Demographic and lifestyle information

Socio-demographic and lifestyle characteristics included age, gender, educational and occupational level, smoking, and alcohol drinking habits. Educational level was categorized as (1) low (primary/secondary), (2) medium (high school), and (3) high (university). Occupational level was categorized as (1) low (unskilled/unemployed workers), (2) medium (partially skilled workers), and (3) high (skilled workers). Physical activity level was categorized as daily (1) low active [expended energy <16.7 kJ (<4 kcal)/min], (2) moderately active [expended energy 16.7–29.3 kJ (4–7 kcal)/min], and (3) highly active [expended energy >29.3 kJ (7 kcal)/min]. Smoking status was categorized as (1) non-smoker and (2) current smoker. Alcohol consumption was categorized as (1) none or moderate drinker (<12 g/day) and (2) alcohol drinker (>12 g/day).

### Dietary assessment

Dietary data were collected by using a food frequency questionnaire (FFQ) based on the tool developed by Willett et al. [28] and subsequently adapted in the Whitehall II Study [29]. The FFQs consisted of 148 food and drink items. An instruction manual that included photographs to facilitate the estimation of portion sizes was used. Participants were asked how often, on average, they had consumed that amount of the item during the last 3 months, with nine responses ranging from “never or less than once per month” to “six or more times per day”. Moreover, participants were asked to include additional foods and frequency of consumption by manual entry.

The average beverage consumption was calculated (in mL) by following the standard portion sizes used in the study and then converted in 24-h intake. For the sake of simplicity, we categorized coffee and tea consumption according to standard cup of coffee (150 mL) and tea (250 mL) in (1) <1 cup/day, (2) 1–2 cups/day, and (3) 3 or more cups/day.

### Clinical measurements

The physical examination included measurement of height, weight, waist circumference, and blood pressure using standard procedures [27]. MetS was defined according to the International Diabetes Federation definition, as having central obesity (WC ≥ 90 cm in men and ≥80 cm in women) and any two of the following: (1) triglycerides >150 mg/dL (1.7 mmol/L), or specific treatment for this lipid abnormality; (2) HDL-c <40 mg/dL (1.03 mmol/L) in males, <50 mg/dL (1.29 mmol/L) in females, or specific treatment for this lipid abnormality; (3) SBP >130 or DBP >85 mm Hg, or treatment of previously diagnosed hypertension; and (4) FPG >100 mg/dL (5.6 mmol/L), or previously diagnosed type two diabetes or treatment of previously diagnosed diabetes [30].

### Statistical analysis

Continuous variables are presented as means and standard deviations (SDs), categorical variables as frequencies and percentages. Variables were examined for normality and skewness (Kolmogorov and Levene tests). One-way ANOVA using Bonferroni correction and Kruskal–Wallis test was used for comparisons of continuous variables, and Chi-square test was used for categorical variables. Linear trends across the coffee consumption categories were tested by assigning each participant the median of the category and modeling this value as a continuous variable. Multivariable logistic regression models were performed to assess the relationship between metabolic parameters (BMI, WC, HDL-c, LDL-c, total cholesterol, FPG, SBP, and DBP) as dependent variables and coffee and tea consumption categories as exposure variables by odds ratios (ORs) and 95 % confidence intervals (CIs). Gender-specific analyses were also conducted to take into account the natural differences in body composition and caloric needs between men and women. Endpoints were adjusted for gender, age, educational level, occupational level, physical activity, smoking status, alcohol drinking, and total energy intake. *p* values <0.05 (two-tailed) were considered significant. Analyses were performed using SPSS software v17.0 (Chicago, USA).

## Results

Baseline characteristics of the 8,822 subjects included in the analysis by coffee and tea consumption category are presented in Table 1. Among high coffee consumers (3 or more cups/day), a higher prevalence of female gender, younger age (average 56.8 years), higher total energy intake, and medium–higher educational and occupational level were found. A significant different distribution was found also according smoking status and alcohol drinking (Table 1). Similar trends were found regarding tea consumption with respect to gender (female) and total energy intake (higher mean intake). High tea consumers were also mostly smokers, not alcohol drinkers, and high physically active.

**Table 1:** Demographic and lifestyle characteristics of the study sample by coffee and tea frequency of consumption categories (*n* = 8,821)

	Coffee consumption			*P*	Tea consumption			*P*
	<1 cup/day	1–2 cups/day	>2 cups/day		<1 cup/day	1–2 cups/day	>2 cups/day	
Total population, *n* (%)	2,734 (31)	3,695 (41.9)	2,392 (27.1)	–	1,743 (19.8)	5,145 (58.3)	1,933 (21.9)	–
Gender, *n* (%)				0.004				0.006
Male	1,399 (51.2)	1,736 (47.0)	1,156 (48.3)		804 (46.1)	2,494 (48.5)	993 (51.4)	
Female	1,335 (48.8)	1,959 (53.0)	1,236 (51.7)		939 (53.9)	2,651 (51.5)	940 (48.6)	
Age (years), mean (SD)	58.2 (7.0)	58.0 (7.0)	56.8 (6.9)	<0.001	57.8 (6.9)	57.7 (7.0)	57.6 (6.9)	0.654
Total energy intake (kcal), mean (SD)	2,106.2 (648.9)	2,127.7 (610.7)	2,233.4 (661.4)	<0.001	2,086.6 (675.6)	2,133.3 (616.5)	2,250.2 (651.5)	<0.001
Educational level, *n* (%)				0.047				0.200
Low	309 (11.3)	448 (12.1)	231 (9.7)		211 (12.1)	548 (10.7)	229 (11.9)	
Medium	1,620 (59.3)	2,197 (59.5)	1,437 (60.1)		1,017 (58.4)	3,113 (60.6)	1,124 (58.2)	
High	801 (29.3)	1,050 (28.4)	722 (30.2)		514 (29.5)	1,480 (28.8)	579 (30.0)	
Occupational level, *n* (%)				0.034				0.449
Low	1,441 (54.5)	1,929 (53.5)	1,224 (52.4)		904 (53.2)	2,678 (53.5)	1,012 (53.8)	
Medium	892 (33.7)	1,258 (34.9)	875 (37.5)		588 (34.6)	1,789 (35.7)	648 (34.4)	
High	313 (11.8)	421 (11.7)	235 (10.1)		207 (12.2)	540 (10.8)	222 (11.8)	
Current smoking (yes), *n* (%)	2,022 (74.1)	2,612 (70.9)	1,519 (63.7)	<0.001	1,172 (67.4)	3,606 (70.3)	1,375 (71.2)	0.028
Alcohol drinking (yes), *n* (%)	127 (4.6)	131 (3.5)	121 (5.1)	0.010	120 (6.9)	182 (3.5)	77 (4.0)	<0.001
Physical activity level, *n* (%)				0.657				0.015
Low	729 (28.1)	1,039 (29.6)	632 (27.9)		477 (29.0)	1,420 (29.2)	503 (27.1)	
Moderate	950 (36.7)	1,264 (36.0)	832 (36.7)		606 (36.8)	1,799 (37.0)	641 (34.6)	
High	912 (35.2)	1,209 (34.4)	800 (35.3)		563 (34.2)	1,647 (33.8)	711 (38.3)	

When examining the association between coffee and tea consumption and various anthropometric measures, a significant inverse trend across categories of coffee consumption was found for BMI (*P* < 0.001), WC (*P* < 0.001), SBP (*P* < 0.001), DBP (*P* < 0.001), triglycerides (*P* = 0.014), and a direct association with HDL-c (*P* = 0.024) and LDL-c (*P* = 0.045) (Table 2). In contrast, significant inverse trend was found for categories of tea drinking and only BMI (*P* < 0.001) and WC (*P* = 0.036) (Table 2).

**Table 2:** Anthropometric characteristics and biomarkers of metabolic syndrome of the study sample by categories of coffee and tea consumption

	Coffee consumption			*P* for trend	Tea consumption			*P* for trend
	<1 cup/day	1–2 cups/day	>2 cups/day		<1 cup/day	1–2 cups/day	>2 cups/day	
Body mass index, mean (SD)	28.25 (4.58)	28.20 (4.59)	27.77 (4.61)	<0.001	28.55 (4.84)	28.05 (4.55)	27.84 (4.47)	<0.001
Waist circumference (cm), mean (SD)	93.34 (12.46)	92.43 (12.24)	91.62 (12.43)	<0.001	93.20 (12.60)	92.31 (12.26)	92.32 (12.45)	0.036
Systolic blood pressure (mmHg), mean (SD)	139.59 (21.15)	138.41 (21.26)	136.06 (20.77)	<0.001	138.49 (21.28)	137.74 (21.29)	138.87 (20.58)	0.531
Diastolic blood pressure (mmHg), mean (SD)	86.83 (12.05)	86.26 (11.68)	85.31 (11.61)	<0.001	86.37 (11.87)	85.87 (11.75)	86.85 (11.81)	0.176
Glucose (mmol/L), mean (SD)	5.39 (1.45)	5.37 (1.44)	5.32 (1.92)	0.081	5.41 (1.49)	5.33 (1.39)	5.40 (2.08)	0.813
Total cholesterol (mmol/L), mean (SD)	5.77 (1.07)	5.83 (1.06)	5.83 (1.05)	0.072	5.84 (1.08)	5.81 (1.05)	5.80 (1.06)	0.290
HDL-cholesterol (mmol/L), mean (SD)	1.43 (0.37)	1.45 (0.39)	1.46 (0.37)	0.024	1.45 (0.39)	1.44 (0.37)	1.44 (0.38)	0.202
LDL-cholesterol (mmol/L), mean (SD)	3.61 (0.95)	3.65 (0.95)	3.66 (0.94)	0.045	3.65 (0.95)	3.64 (0.95)	3.64 (0.96)	0.755
Triglycerides (mmol/L), mean (SD)	1.60 (0.77)	1.59 (0.76)	1.55 (0.74)	0.014	1.60 (0.75)	1.58 (0.75)	1.57 (0.78)	0.290

Both coffee and tea consumption were inversely associated with having MetS (Tables 3, 4). The multivariable regression analysis of overall sample, adjusted for gender, age, educational level, occupational level, physical activity, smoking status, alcohol drinking, total energy intake, and tea consumption, revealed that high coffee consumption (>2 vs. <1 cup/day) was significantly associated with several components of MetS, such as WC, blood pressure cut offs, and triglycerides (Table 3). When the analysis was repeated by gender, coffee consumption was inversely related to WC in men, but not in women, whereas among the latter a significant association with hypertension and low HDL-c was found (Table 3). Results about tea consumption were slightly different (Table 4). Despite the high consumption of tea was inversely related with having MetS, the analysis stratified by gender revealed a significant association for men, but not for women. The strongest relation was between tea consumption and central obesity, with a trend over categories of tea consumption in both men and women. Finally, tea consumption was also inversely related to FPG in women, but not in men.

**Table 3:** Multivariable adjusted odds ratios (95 % confidence interval)^a^ for metabolic syndrome and its individual components by categories of coffee consumption, overall and by gender

	Coffee consumption					
	Men, *n* (%)	OR (95 % CI)^a^	Women, *n* (%)	OR (95 % CI)^a^	Overall, *n* (%)	OR (95 % CI)^a^
Metabolic syndrome (yes)						
<1 cup/day	397 (28.4)	1	429 (32.1)	1	826 (30.2)	1
1–2 cups/day	461 (26.6)	0.93 (0.78–1.10)	608 (31.0)	0.93 (0.79–1.10)	1,069 (28.9)	0.92 (0.82–1.04)
>2 cups/day	268 (23.2)	0.82 (0.67–0.99)	298 (24.1)	0.69 (0.57–0.83)	566 (23.7)	0.75 (0.66–0.86)
* P* for trend		0.003		<0.001		<0.001
Waist circumference ≥90 cm in men and ≥80 cm in women						
<1 cup/day	460 (32.9)	1	625 (46.8)	1	1,085 (39.7)	1
1–2 cups/day	512 (29.5)	0.85 (0.72–1.00)	904 (46.1)	1.02 (0.88–1.19)	1,416 (38.3)	0.93 (0.83–1.04)
>2 cups/day	316 (27.3)	0.81 (0.68–0.98)	524 (42.4)	0.90 (0.76–1.07)	840 (35.1)	0.86 (0.75–0.97)
* P* for trend		0.002		0.210		0.001
Systolic blood pressure ≥130 mmHg or diastolic blood pressure ≥85 mmHg or hypertensive treatment						
<1 cup/day	963 (68.8)	1	802 (60.1)	1	1,765 (64.6)	1
1–2 cups/day	1,140 (65.7)	0.89 (0.75–1.04)	1,112 (56.8)	0.92 (0.79–1.08)	2,252 (60.9)	0.90 (0.80–1.01)
>2 cups/day	727 (62.9)	0.88 (0.74–1.06)	597 (48.3)	0.70 (0.59–0.83)	1,324 (55.4)	0.78 (0.69–0.88)
*P* for trend		0.002		<0.001		<0.001
HDL-cholesterol <40 mg/dL in men and <50 mg/dL in women						
<1 cup/day	265 (18.9)	1	350 (26.2)	1	615 (22.5)	1
1–2 cups/day	335 (19.3)	1.01 (0.84–1.22)	503 (25.7)	0.93 (0.79–1.10)	838 (22.7)	0.97 (0.85–1.10)
>2 cups/day	220 (19.0)	1.02 (0.83–1.26)	282 (22.8)	0.78 (0.65–0.95)	502 (21.0)	0.88 (0.77–1.02)
*P* for trend		0.942		0.049		0.210
Tryglicerides ≥150 mg/dL						
<1 cup/day	669 (47.8)	1	614 (46.0)	1	1,283 (46.9)	1
1–2 cups/day	871 (50.2)	1.13 (0.98–1.31)	877 (44.8)	0.95 (0.82–1.11)	1,748 (47.3)	1.03 (0.93–1.15)
>2 cups/day	542 (46.9)	0.96 (0.81–1.13)	459 (37.1)	0.77 (0.64–0.91)	1,001 (41.8)	0.86 (0.76–0.97)
*P* for trend		0.718		<0.001		<0.001
Fasting plasma glucose ≥100 mg/dL or diabetes treatment						
<1 cup/day	169 (12.1)	1	113 (8.5)	1	282 (10.3)	1
1–2 cups/day	204 (11.8)	0.94 (0.74–1.18)	180 (9.2)	1.10 (0.84–1.43)	384 (10.4)	0.99 (0.83–1.18)
>2 cups/day	125 (10.8)	0.97 (0.75–1.26)	74 (6.0)	0.80 (0.58–1.11)	199 (8.3)	0.90 (0.73–1.10)
*P* for trend		0.328		0.250		0.020

^a^Adjusted for gender (except when analyses were stratified by sex), age, educational level, occupational level, physical activity, smoking status, alcohol drinking, total energy intake, and tea consumption

**Table 4:** Multivariable adjusted odds ratios (95 % confidence interval)^a^ for metabolic syndrome and its individual components by categories of tea consumption, overall and by gender

	Tea consumption					
	Men, *n* (%)	OR (95 % CI)^a^	Women, *n* (%)	OR (95 % CI)^a^	Overall, *n* (%)	OR (95 % CI)^a^
Metabolic syndrome (yes)						
<1 cup/day	242 (30.1)	1	295 (31.4)	1	537 (30.8)	1
1–2 cups/day	637 (25.5)	0.79 (0.65–0.95)	777 (29.3)	0.89 (0.75–1.07)	1,414 (27.5)	0.84 (0.74–0.96)
>2 cups/day	247 (24.9)	0.75 (0.60–0.93)	263 (28.0)	0.82 (0.65–1.01)	510 (26.4)	0.79 (0.67–0.92)
*P* for trend		0.017		0.042		0.003
Waist circumference ≥90 cm in men and ≥80 cm in women						
<1 cup/day	269 (33.5)	1	472 (50.3)	1	741 (42.5)	1
1–2 cups/day	731 (29.3)	0.78 (0.65–0.93)	1,186 (44.7)	0.80 (0.68–0.95)	1,917 (37.3)	0.79 (0.70–0.90)
>2 cups/day	288 (29.0)	0.73 (0.59–0.90)	395 (42.0)	0.71 (0.58–0.87)	683 (35.3)	0.73 (0.63–0.84)
*P* for trend		0.052		<0.001		<0.001
Systolic blood pressure ≥130 mmHg or diastolic blood pressure ≥85 mmHg or hypertensive treatment						
<1 cup/day	546 (67.9)	1	535 (57.0)	1	1,081 (62.0)	1
1–2 cups/day	1,597 (64.0)	0.85 (0.71–1.02)	1,452 (54.8)	0.89 (0.75–1.05)	3,049 (59.3)	0.87 (0.77–0.99)
>2 cups/day	687 (69.2)	1.09 (0.88–1.36)	524 (55.7)	0.96 (0.78–1.17)	1,211 (62.6)	1.02 (0.88–1.18)
*P* for trend		0.415		0.592		0.614
HDL-c <40 mg/dL in men and <50 mg/dL in women						
<1 cup/day	163 (20.3)	1	215 (22.9)	1	378 (21.7)	1
1–2 cups/day	470 (18.8)	0.92 (0.75–1.14)	690 (26.0)	1.18 (0.98–1.42)	1,160 (22.5)	1.06 (0.93–1.22)
>2 cups/day	187 (18.8)	0.91 (0.71–1.17)	230 (24.5)	1.03 (0.82–1.29)	417 (21.6)	0.99 (0.84–1.17)
* P* for trend		0.466		0.432		0.902
Tryglicerides ≥150 mg/dL						
<1 cup/ cup/day	414 (51.5)	1	392 (41.7)	1	806 (46.2)	1
1–2 cups/day	1,202 (48.2)	0.92 (0.78–1.09)	1,160 (43.8)	1.11 (0.94–1.31)	2,362 (45.9)	1.01 (0.90–1.13)
>2 cups/day	466 (46.9)	0.86 (0.71–1.05)	398 (42.3)	1.01 (0.83–1.23)	864 (44.7)	0.93 (0.81–1.07)
*P* for trend		0.060		0.795		0.339
Fasting plasma glucose ≥100 mg/dL or diabetes treatment						
<1 cup/day	96 (11.9)	1	94 (10.0)	1	190 (10.9)	1
1–2 cups/day	296 (11.9)	0.96 (0.74–1.24)	203 (7.7)	0.74 (0.56–0.97)	499 (9.7)	0.85 (0.71–1.03)
>2 cups/day	106 (10.7)	0.87 (0.64–1.19)	70 (7.4)	0.70 (0.49–0.99)	176 (9.1)	0.79 (0.63–1.00)
*P* for trend		0.378		0.042		0.070

^a^Adjusted for gender (except when analyses were stratified by sex), age, educational level, occupational level, physical activity, smoking status, alcohol drinking, total energy intake, and coffee consumption

## Discussion

In the present study, we evaluated the relationship between coffee and tea consumption with cardiovascular risk factors and MetS in a large sample of men and women living in Krakow, Poland. Both coffee and tea consumption were negatively associated with MetS. The overall prevalence of MetS in the sample was 27.9 %, similar to previous reports from Poland [31–33]. The proportion of MetS in low and high consumers of coffee was 30.2 and 23.7 %, respectively. Similar differences were found for tea consumers, for instance, 30.8 % among low consumers and 26.4 % in high consumers.

The relationship between coffee consumption and MetS has not yet been well investigated, and results of previous epidemiological studies are contrasting. Several studies reported that coffee consumption was inversely associated with MetS in the Japanese population [20–22]. On the other hand, studies conducted in central [23, 24] and north [25] European countries reported no relation between coffee consumption and the development of MetS or its components. In contrast, a previous study conducted in a Southern European region (namely the Mediterranean area) showed a negative (beneficial) association [26]. Epidemiological studies exploring the favorable effect of tea consumption on MetS have reported more univocal results, such as an inverse association [34–36] and others even a therapeutic effect [37–39]. We reported an overall negative association between coffee and tea consumption and MetS, but contrarily to the previous studies, and this association was more remarked for coffee, rather than for tea. The components of MetS that have been found to be more susceptible to consumption of coffee and tea were primarily central obesity, with no differences between men and women, whereas association with cholesterol and glucose metabolisms greatly depended on the type of beverages and gender. Our results confirm in part the findings reported in previous studies assessing that habitual consumptions of tea were favorably associated with triglycerides [40], FPG [41, 42] and body fat [43], whereas coffee was more likely associated with improvements in glucose and insulin metabolism [44] as well as with BMI and WC [45].

Although the exact mechanism through which coffee and tea may prevent MetS is still unclear, previous studies suggested that their healthy effects on chronic diseases may depend on the antioxidant compounds contained in both coffee and tea [46]. These beverages contain significant amounts of vitamins and minerals, such as ascorbic acid (vitamin C), several B vitamins, riboflavin, niacin, folic acid, pantothenic acid, magnesium, potassium, manganese, and fluoride [47, 48]. Among other bioactive compounds, polyphenols are among the best candidates to be responsible for the beneficial actions of coffee and tea consumption on various metabolic disorders [49]. The polyphenols found in coffee are hydroxycinnamic acids (such as caffeic and ferulic acids), among which the most effective against CVD have been reported to be isomers of chlorogenic acid [17]. The main polyphenols contained in tea belong to the family of the catechins, flavanols, flavanol glycosides, flavandiols, and depsides [18]. The beneficial effects of polyphenols contained in coffee and tea in preventing chronic diseases (especially CVD) could be due to their ability to ameliorate endothelial function [50], suppress vascular endothelial cell expression of pro-inflammatory cytokines [51], and consequent upregulation of adhesion molecules and monocyte adhesion [52]. Among the other effects, tea has been demonstrated to ameliorate insulin resistance [53], whereas coffee has been associated with adiponectin levels [54]. However, differences in type and content of polyphenols depending on quality of both coffee and tea may result in a stronger beneficial effect of one rather than the other and this issue needs to be investigated further.

Some mechanisms to explain the healthful effects related to caffeine on preventing the MetS have been hypothesized, including sympathetic over activation, antagonism of adenosine receptors, increased norepinephrine release, a sympathomimetic agent that is capable of increasing energy expenditure, and promoting the loss of body fat [55, 56]. On the other hand, other studies failed in to demonstrate such beneficial effects and reported that caffeine may explicate itself favorable effects on the various components of the MetS [57].

Our study has some limitations that should be addressed. First, because of its cross-sectional nature, the associations retrieved in the study do not indicate causality. Reverse causation should be taken into account when exploring behavioral choices that may be influenced by health status. Also, coffee and tea consumption could be influenced by other lifestyle characteristics and not be equally distributed by social status. Adjustment for smoking status and education, which was done in our analysis, could not be sufficient to control the confounding effect of background characteristics. Second, the question regarding tea consumption was not specific for different types of tea. Despite the participants were allowed to add supplementary foods, such as green and herbal teas, the most of them did not differentiate between types of tea, thus not allowing us to assess possible differences on effects. Moreover, dietary information was self-reported, which may have led to recall bias. Finally, health information of individuals not included in the analysis due to lack of blood samples should be taken into account when considering our results. However, despite these limitations, our findings remain of significant value, since this was the first study describing the relationship between consumption of coffee and tea and components of MetS in a large sample from East Europe.

In conclusion, a significant negative association between coffee consumption and prevalence of MetS both in men and in women was found, whereas tea consumption was associated with MetS only in men. High coffee and tea consumption were also associated with a decreased prevalence of central obesity and better cholesterol and glucose metabolism. These findings reinforce the hypothesis on the possible health benefits of polyphenols. Due to methodological limitations, we cannot exclude that the observed associations on coffee/tea and MS are due to other healthy lifestyle behaviors, and further prospective studies are needed to better adjust for potential confounding factors.
